# Modifications in ocular microperfusion after transcatheter aortic valve implantation

**DOI:** 10.1038/s41598-023-41054-z

**Published:** 2023-08-30

**Authors:** Anne Caroline Wolpers, Thomas Welchowski, Alexander Sedaghat, Maximilian W. M. Wintergerst, Baravan Al-Kassou, Robert P. Finger, Jan Henrik Terheyden

**Affiliations:** 1 Department of Medicine II, Heart Center, University Hospital Bonn, Bonn, Germany; 2 Department of Cardiology and Internal Intensive Care, Asklepios Clinic St. Georg, Hamburg, Germany; 3 Institute for Medical Biometry, Informatics and Epidemiology, University of Bonn, Bonn, Germany; 4 RheinAhrCardio Practice for Cardiology, Wilhelmstr. 14, 53474 Bad Neuenahr-Ahrweiler, Germany; 5 Department of Ophthalmology, University Hospital Bonn, Venusberg-Campus 1, 53127 Bonn, Germany; 6 Department of Ophthalmology, University Hospital Mannheim & Medical Faculty Mannheim, University of Heidelberg, Theodor-Kutzer-Ufer 1-3, 68167 Mannheim, Germany

**Keywords:** Prognostic markers, Valvular disease

## Abstract

Cerebral embolization is a known complication of transcatheter aortic valve implantation (TAVI) but the effect of the procedure on the ocular perfusion is currently unclear. Thus, we investigated post-procedural morphologic and perfusion changes of the retina and choroid, using optical coherence tomography angiography (OCTA) and color fundus photography (CFP) in a prospective cohort study. Ophthalmic examinations were conducted pre- and post-TAVI. OCTA images were analyzed quantitatively based on vessel density and skeleton density of the superficial and deep retinal plexus as well as the signal intensity and flow deficits in the choriocapillaris. CFP images were assessed for presence of acute retinal ischemia, optic nerve swelling, vessel emboli, hemorrhages and cotton wool spots. Data was analyzed using linear mixed models. Twenty patients (9 women; 11 men) at a mean age of 81 ± 6 years were included. Pre- and post-interventional ocular imaging data were available for 32 eyes. The analysis revealed a significant impairment of the choriocapillaris perfusion after TAVI with an increased proportion of flow deficits (*p* = 0.044). When controlling for blood pressure, the average size of choriocapillaris flow voids was significantly increased (systolic and diastolic, *p* = 0.039 and 0.029). Qualitatively, focal areas of retinal ischemia were detected on OCTA in 33% of participants. Silent emboli or cotton wool spots were identified on CFP in 21%. Our findings indicate a reduced choroidal perfusion as well as areas of retinal ischemia and embolization in a considerable proportion of patients following TAVI. Pending confirmation in a larger sample, these complications merit monitoring as well as inclusion in consent procedures for TAVI.

## Introduction

Transcatheter aortic valve implantation (TAVI)—although highly effective^1–3^—is associated with both clinical and subclinical cerebral emboli. Post-procedural stroke is a potential sequel of this embolization and known to be a strong predictor of early and long-term mortality after TAVI procedures^4^. In fact, cerebral magnet resonance imaging (MRI) detects cerebral emboli in approximately 75% of patients undergoing TAVI—with the majority of these emboli being clinically silent^5–7^.

Given the shared vascular supply of eye and brain structures, there is also evidence of ocular microembolization in fundus examination after TAVI in 15% of a published cohort^8^. While traditional fundus examination allows direct visualization of arterioles and venules, optical coherence tomography (OCT) and OCT angiography (OCTA) enable a non-invasive and precise assessment, stratification, and quantification of the retinal and choroidal structure and microperfusion at a capillary level^9^ . Previous studies using these techniques identified changes in retinal thickness and flow^10,11^. However, choroidal perfusion has not been investigated for a comparable cohort so far. Moreover, flow alterations were only assessed qualitatively, whereas availability of an ophthalmologist may limit the real-world use of oculomics after TAVI in asymptomatic patients. We have thus used both OCTA and fundus photographs with qualitative and automated quantitative methods to assess the eye’s microvasculature and perfusion in a cohort of patients undergoing TAVI, hypothesizing that the intervention leads to an impairment in the retinal or choroidal flow as a possible effect of silent ocular embolization.

## Methods

### Patient recruitment

Prior to patient recruitment, approval was obtained from the ethics committee of the University of Bonn (Approval ID AZ 046/21). Inpatients with planned TAVI due to symptomatic high-grade aortic valve stenosis were prospectively recruited during outpatient visits at the department of cardiology, University Hospital Bonn, Germany and examined pre- and post-TAVI. During the two study visits per participant, an eye assessment and blood pressure measurements were conducted. The eye assessment included best-corrected visual acuity (BCVA), intraocular pressure (IOP), slit lamp and fundus examination, as well as OCTA imaging and color fundus photography. More details on the ocular imaging protocol are given below. All participants’ pupils were dilated using 0.5% tropicamid eye drops before the images were obtained.

Prior to TAVI, the patients had the following routine assessments performed: Transthoracic and transesophageal echocardiography, computer tomography (CT)-visualization of the aorta, the aortic valve and annulus, pulmonary function, duplex sonography of the carotid arteries and peripheral arteries and laboratory testing. The results of these assessments were discussed with a multidisciplinary team of cardiologists and heart surgeons before listing for a TAVI procedure. The TAVI procedure with a balloon-expanding or self-expanding valve has been previously described^1,12^. Cerebral embolic protection was not used, following the current guideline recommendations^13,14^.

The study adhered to the principals of the Declaration of Helsinki and all participants of the study gave written informed consent prior to participation. Exclusion criteria were a reduction of image quality (e.g., relevant shadow artifacts, OCTA signal strength index < 7), ocular conditions confounding the interpretation of images, instability to fixate, and systemic conditions not allowing participants to sit steadily or be transported to the imaging unit (i.e., immobility).

### Ocular image acquisition

As outlined above, non-invasive ocular imaging was performed at two independent visits, which took place before and after the TAVI procedure. The first study visit took place less than two months before TAVI (1–54 days, median 1 day) and the second study visit was conducted fewer than ten days (2–8 days, median 3 days) after TAVI. Microvascular flow information was acquired using a swept-source OCTA device with a central wavelength of 1040–1060 nm and recorded 100,000 A-scans per second (PLEX Elite 9000, Carl Zeiss Meditec, Dublin, California). Two scans were performed per eye (3 × 3 mm cube scan of the macula in all participants and 15 × 9 mm wide-field scan centered at the posterior pole in a subsample). Subsequently, the proprietary automatic segmentation tool was used on the 3 × 3 mm cube scans and en face images of the superficial retinal vessel layer (flow in retinal nerve fiber layer, ganglion cell layer and inner plexiform layer), deep retinal layer (flow in inner nuclear layer, outer plexiform layer and Henle fiber layer) and choriocapillaris layer (flow between 10 and 40 µm below retinal pigment epithelium-fit segmentation) were generated. Superficial vessel projections were removed from the deep retinal layer and choriocapillaris en face images, using an algorithm integrated in the device. In addition, fundus photographs focused on the posterior pole were acquired with a wide-field camera (Clarus 500, Carl Zeiss Meditec, Dublin, California) in a subsample, which provides a 133° field of view with a single image.

### Image analysis

We used Fiji, an open source software based on ImageJ, version 1.51w to quantify perfusion of the retinal layers and the choriocapillaris per eye based on the acquired OCTA cube scans. Vessel structures in the superficial and deep retinal layers were binarized, using an automated algorithm^15^. In a previous study investigating different thresholding techniques, the Otsu algorithm generated highly consistent results, with intra-class correlation coefficients > 0.9 in repeated scans^16^. The parameters vessel densitiy (VD) and vessel skeleton density (SD) were calculated as previously defined^17^. Skeleton density is independent of vessel diameters and therefore more sensitive to changes of the capillaries. To analyze alterations of the choriocapillaris, the mean and standard deviation of the raw signal intensity and flow voids were analyzed as previously established^18,19^. Flow voids were defined as coherent areas of perfusion below a threshold determined by the Phansalkar binarization algorithm (radius 8 px)^20^, exceeding the intercapillary distance^19^. We considered the individual number of flow signal voids per eye, the average size of these flow voids and the proportion of total scan area occupied by flow voids for our analysis (“flow deficits”). As an additional structural outcome, subfoveal choroidal thickness was measured in a single horizontal swept-source OCTA B-scan by an ophthalmologist, using proprietary software on the OCTA device.

Fundus photographs were graded for the onset of acute retinal ischemia, optic nerve swelling, vessel emboli, hemorrhages, and cotton wool spots by an ophthalmologist. Wide-field OCTA images were graded for the onset of retinal ischemic areas after TAVI by an ophthalmologist.

### Statistical analysis

Statistical analysis was conducted with R version 4.1.2 (R Core Team, Vienna, Austria) and IBM SPSS statistics (version 27, IBM Corporation, Armonk, NY). Continuous variables were reported with mean value and standard deviation. Categorical variables were reported as frequencies and percentages. The Wilcoxon test was used to compare the changes in post-procedural parameters.

OCTA flow parameters and choroidal thickness were analysed using mixed-effects models. We included the examination (pre- or post-TAVI) as the dependent variable and one ophthalmic variable per model as an independent variable (fixed effect) as well as a random intercept per patient, to correct for the intercorrelation of paired eye data. With this method, we were able to include all data points from our cohort in the analysis. Additional mixed models corrected for the known impact of individual blood pressure on ocular blood flow parameters^21,22^. These models included systolic or diastolic as an additional independent variable (fixed effect) besides the respective ophthalmic variable. *P*-values < 0.05 were considered statistically significant.

## Results

Twenty-eight patients completed the imaging protocols pre- and post-TAVI. After evaluation of the images and exclusion of eight individuals not meeting the inclusion criteria, twenty participants (32 single eyes with sufficient image quality) could be included. Reasons for exclusion after recruitment were a loss to follow-up in four patients (Covid-19, n = 2; postoperative delirium, 1; withdrawal of consent, 1), and insufficient image quality due to an instable fixation in the remaining four individuals.

**Figure 1:**
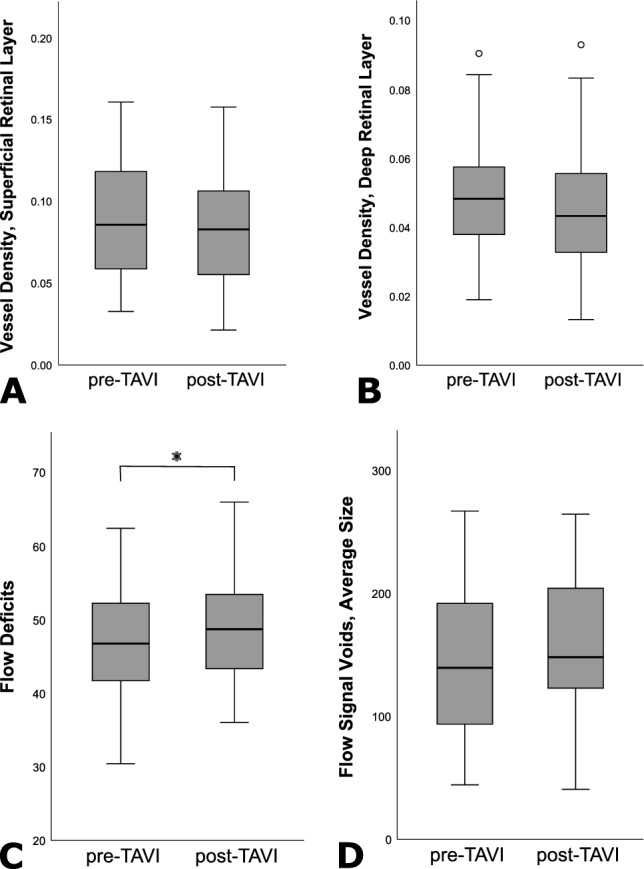
Perfusion of the retinal and choriocapillaris microvasculature pre- and post- transcatheter aortic valve implantation (TAVI), measured by optical coherence tomography angiography of the macula. Values over 1.5 interquartile range below the first quartile or above the third quartile were defined as outliers. The displayed data include data from both eyes.

**Figure 2:**
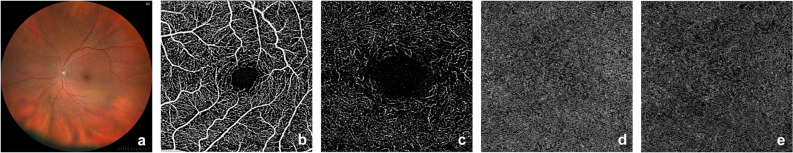
Imaging modalities implemented in the study in an exemplary participant: Color fundus photography (**a**), and optical coherence tomography angiography (**b**–**e**); superficial retinal plexus, deep retinal plexus and choriocapillaris en face projections), pre-TAVI (**a**–**d**) and post-TAVI (**e**). Choriocapillaris flow deficits are focal, dark areas in panels d and e.

Participants were on average 81 ± 6 years old and 45% were women (Table 1). Prior to TAVI, most participating patients were symptomatic with New York Heart Association (NYHA) III dyspnea. The majority of participants was on anticoagulation or antiplatelet drugs. The type of valve (balloon-expanding versus self-expanding) implanted during TAVI was equally distributed in our cohort (Table 1). The ejection fraction as assessed by echocardiography significantly improved after TAVI (Table 2). With regard to the ocular perfusion after TAVI, we detected significant changes in the perfusion of the choriocapillaris layer, the innermost layer of the choroid (Table 3; Fig. 1). The mean choriocapillaris signal intensity detected by OCTA decreased after the procedure and the size of existing flow voids as well as the total area of non-perfused choriocapillaris en face showed a significant increase. The trend of increased flow voids remained stable when correcting for the impact of blood pressure on the choriocapillaris perfusion. Figure 2 shows pre- and post-TAVI images from an exemplary patient. Unlike choriocapillaris perfusion, retinal perfusion was not significantly impaired after TAVI in this quantitative analysis. Structurally, subfoveal choroidal thickness increased from a median of 209 µm pre-TAVI to a median of 218 µm post-TAVI but this did not reach the level of statistical significance (uncorrected model: β = 1.01, *p* = 0.82; corrected for systolic blood pressure: β = 1.02, *p* = 0.46; corrected for diastolic blood pressure: β = 1.03, *p* = 0.22).

**Table 1:** Baseline characteristics of patient cohort (n = 20).

	Mean ± SD or n(%)
Age, years	81 ± 6
Gender	
Female (%)	9 (45)
Male (%)	11 (55)
Body mass index, kg/m^2^	28 ± 5
NYHA class	3 ± 1
Aortic valve area, cm^2^	0.76 ± 0.13
EuroSCORE II, %	4.6 ± 4.4
STS Score; %	3.8 ± 3.2
BCVA, logMAR	
Right eye	0.2 ± 0.1
Left eye	0.1 ± 0.1
Intraocular pressure, mmHg	
Right eye	16 ± 3
Left eye	17 ± 3
Systemic comorbidities	
Hypertension (%)	16 (80)
Diabetes mellitus (%)	6 (30)
Dyslipidemia (%)	13 (65)
Chronic obstructive pulmonary disease (%)	3 (15)
Prior stroke or TIA (%)	2 (10)
Atrial fibrillation or flutter (%)	8 (40)
CHA_2_DS_2_-Vasc-Score	4.6 ± 1.5
Peripheral artery disease (%)	8 (40)
Carotid artery disease (%)	7 (35)
Extracardiac arteriopathy (%)	9 (45)
Coronary artery disease (%)	14 (70)
Procedural characteristics	
Prosthetic valve type	
Balloon-expanding (%)	10 (50)
Self-expanding (%)	10 (50)
Prosthesis size, mm diameter	27 ± 2
Predilatation (%)	19 (95)
Postdilatation (%)	1 (5)
Antithrombotic medication	
Oral Anticoagulation	9 (45)
Acetylsalicylic acid	11 (55)
Other antiplatelet, i.e. clopidogrel, prasugrel, ticagrelor	14 (70)
‚Dual Therapy ‘	3 (15)
Dual antiplatelet therapy	11 (55)

Parameters are presented as frequencies n (percentage) or quantity ± standard deviation.

NYHA: New York Heart Association, class to describe extent of symptomatic heart failure; EUROScore II and STS score: scores to estimate the perioperative risk before cardiac surgery helping to decide between interventional and operative therapy; CHA_2_DS_2_-Vasc-Score: score estimating risk of Stroke in atrial fibrillation or flutter; ‚Dual Therapy‘: combination of an anticoagulant and platelet aggregation inhibitor.

**Table 2:** Circulatory changes after transcatheter aortic valve implantation (TAVI).

	Pre-TAVI	Post-TAVI	*p*-Value
Ejection fraction, %	55 ± 5	60 ± 7	0.004
Systolic BP, mmHg	137 ± 19	130 ± 18	0.21
Diastolic BP, mmHg	73 ± 7	69 ± 8	0.19
GFR (estimation), ml/min	58 ± 15	62 ± 14	0.75

Values are presented as mean ± standard deviation (measurement unit).

*BP* blood pressure, *GFR* glomerular filtration rate.

**Table 3:** Mixed-effect models of changes in the microperfusion of the retina and the choriocapillaris after transcatheter aortic valve implantation (TAVI), assessed by optical coherence tomography angiography.

	Uncorrected		Systolic BP-corrected		Diastolic BP-corrected	
	β [95% CI]	*p*	β [95% CI]	*p*	β [95% CI]	*p*
Superficial retinal layer						
Vessel density	0.888 [0.785;1.005]	0.067	0.932 [0.818;1.063]	0.298	0.955 [0.828;1.101]	0.527
Vessel skeleton density	0.940 [0.884;1.000]	0.055	0.966 [0.905;1.030]	0.298	0.974 [0.907;1.046]	0.470
Deep retinal layer						
Vessel density	0.895 [0.797;1.005]	0.067	0.926 [0.817;1.049]	0.234	0.954 [0.834;1.090]	0.492
Skeleton density	0.954 [0.905;1.006]	0.090	0.972 [0.918;1.029]	0.334	0.984 [0.926;1.046]	0.608
Choriocapillaris						
Flow deficits (%)	1.057 [1.003;1.113]	0.044*	1.042 [0.985;1.102]	0.155	1.033 [0.973;1.098]	0.290
Flow signal voids (number)	1.204 [0.895;1.622]	0.227	1.309 [0.962;1.784]	0.093	1.281 [0.919;1.786]	0.149
Flow signal voids (average size)	1.128 [0.957;1.328]	0.160	1.196 [1.016;1.408]	0.039*	1.226 [1.028;1.463]	0.029*

*BP* blood pressure. *P*-values marked with an asterisk were statistically significant.

Gradable fundus photographs from both visits were available in a subsample of fourteen participants (70%) and gradable 15 × 9 mm OCTA images in twelve participants (60%). On fundus photography, signs of embolization were detectable in three participants (21% of subsample). A single, new-onset retinal vessel embolus was found in three participants but no adjacent areas of ischemia or hemorrhages after TAVI were identified. Furthermore, a single cotton wool-spot after TAVI was found in one of these participants. None of the participants showed optic nerve swelling following TAVI. On wide-field OCTA, new-onset areas of focal retinal ischemia not visible on color fundus photography were detected in four participants (33% of subsample). Most of these areas were located outside the area covered by the 3 × 3 mm OCTA scan. Overall, the average size of flow signal voids increased more in participants with signs of embolization in fundus photography or with new-onset areas of retinal ischemia detected on wide-field OCTA (mean increase: 27 pixels per flow void in eyes with qualitative changes, 11 pixels in eyes without qualitative changes), while this did not hold true for changes in the overall proportion of flow deficits (median increase: 3.6% and 4.8%, respectively).

## Discussion

We have found the perfusion of the choroid but not the retina significantly reduced after TAVI, which was irrespective of blood pressure. This finding is consistent with peri-interventional embolic lesions of brain tissue, which are known from studies using MRI. We have furthermore identified signs of silent retinal embolization in a noticeable proportion of individuals undergoing TAVI, using color fundus photography and wide-field OCTA. Our results imply a potential use of OCTA as a fast, non-invasive screening tool for embolic material entering the circulation during TAVI. Furthermore, they raise awareness of potential vision-threatening complications related to alterations of both the choroidal and retinal circulations after TAVI and suggest that merits inclusion in consent procedures for TAVI.

Only few studies have been previously published on the use of ophthalmic imaging modalities to detect ocular complications after TAVI. Fusi-Rubano et al. identified retinal embolic events in 15% (3 individuals) of their cohort of patients undergoing TAVI (cotton wool spots or visible emboli), using ophthalmoscopy and fundus photography^8^, which is comparable to a proportion of 21% in our study. Erdöl et al. applied structural optical coherence tomography of the macula to a cohort of patients undergoing TAVI and identified temporary, reversible changes of the total macular thickness and ganglion cell layer thickness^10^. Recently, Gunzinger et al. used the flow-sensitive method of OCTA to investigate changes of the retinal microperfusion after TAVI and identified new-onset capillary dropouts in 29% (8 individuals) of their cohort based on a qualitative methodology similar to ours, most of which were within the vascular arcade or close to the optic disc. This proportion is also supported by the results in our cohort, where retinal capillary drop-outs were qualitatively identified in 33%. Use of an additional, quantitative approach, however, did not reveal any significant changes of the retinal capillaries after TAVI in this cohort, which is also supported by our data^11^.

To the best of our knowledge, we provide the first available evidence of vascular flow alterations in the choriocapillaris after TAVI, i.e. the innermost vessel layer of the choroid, which is essential for the retinal pigment epithelium and photoreceptors. Due to its high perfusion rate, the choroid may represent a particularly promising structure in vascular flow-related oculomics. However, reliable quantification requires modern, high-resolution imaging techniques like OCTA, which allows for non-invasive image acquisition within seconds and renders three-dimensional datasets containing blood flow information including the choriocapillaris. Our results suggest that other local factors such as secondary microemboli or humoral vasoconstrictive factors change the microperfusion in the choroid after TAVI. Since structural choroidal changes were not significantly altered after TAVI, we propose to further investigate particularly OCTA as a modality to monitor multi-organ thrombembolic complications following TAVI. While retinal capillary dropout seems to be associated with ischemic brain lesions on MRI in only 25% of cases^11^, the strong association between flow alterations specific to the choriocapillaris layer and long-term cerebral changes make choriocapillaris flow deficits a promising biomarker^23,24^.

We did not identify any direct vision-threatening complications after TAVI in our cohort. Based on our findings and the above-mentioned reports which represent a total number of 118 TAVI cases^8,10,11^, an increased risk of acute ischemic ocular events such as central retinal artery occlusion, branch retinal artery occlusion and ischemic optic neuropathy as a consequence of TAVI should be suspected. Nevertheless, none of these 118 cases includes a TAVI-related vision-threatening complication and thus, these ocular events are either temporary or have lower incidence rates than manifest strokes secondary to TAVI, where incidences between 0.6 and 6.7%^4,25,26^, with a median around 3%^4^ have been reported.

Strengths of our study include its rigorously characterized cohort of patients, using a multitude of cardiac and ophthalmic assessments, as well as its profound analysis of multimodal imaging data. Changes in the choriocapillaris flow are affected by ocular diseases such as age-related macular degeneration and systemic conditions like arterial hypertension^27–29^. The choriocapillaris flow impairment after TAVI in our cohort was independent of chronic ocular and systemic changes by the design of our study. Also, it could not be explained by changes in blood pressure. Another strength of our study is its homogenous cohort, with participants very typical for an elderly patient population in need of TAVI treatment and a consistent interventional methodology. In agreement with existing data, our cohort showed a significant improvement of the ejection fraction after TAVI^30^. However, our study also comes with limitations. The limited sample size of the cohort did not allow us to investigate how ocular changes relate to other factors, e.g. procedural steps during TAVI, the type of implant, calcium score of the aortic valve, and systemic manifestations of atherosclerosis, and we did not include a control group not undergoing TAVI. Also, we did not correct for multiple comparisons. This is related to the pilot character of our study and suggests additional research on OCTA imaging after TAVI. The limited sample size is likely insufficient to provide precise estimates of ocular complication rates after TAVI but adds to other published cohorts. Due to long travel distances, we were not able to perform longer term follow-up examinations to investigate if the identified microvascular flow changes are only temporary or permanent. Lastly, image quality limitations and mobility restrictions did not allow us to follow-up all participants who were originally recruited for our study, which may have led to attrition bias. With the popularization of more mobile OCTA devices, we expect this to be improved in the future.

In conclusion, this study identified a so far unknown impairment of the choroidal microperfusion in patients undergoing TAVI. Our data also support existing data on silent embolization to the retinal vasculature during TAVI. Future research needs to determine if OCTA may be a useful tool to screen for embolic complications of TAVI earlier than currently possible. Further, OCTA could serve for monitoring of complication rate after TAVI in clinical studies and to detect individuals with a high embolization load and thus a potentially higher long-term mortality rate.

## Data Availability

The data proving the main findings of the study are contained within the manuscript. Additional data are available upon reasonable request from the University Hospital Bonn, Department of Ophthalmology at phone number +4,922,828,719,844.
